# Desipramine induces anti-inflammatory dorsal root ganglion transcriptional signatures in the murine spared nerve injury model

**DOI:** 10.1016/j.ynpai.2024.100153

**Published:** 2024-03-20

**Authors:** Randal A. Serafini, Aarthi Ramakrishnan, Li Shen, Venetia Zachariou

**Affiliations:** a Nash Family Department of Neuroscience, Icahn School of Medicine at Mount Sinai, New York, NY 10029, United States; b Department of Pharmacology, Physiology & Biophysics, Avedisian and Chobanian School of Medicine at Boston University, Boston, MA 02118, United States

**Keywords:** Desipramine, Dorsal root ganglia, Inflammation, Nucleus accumbens, RNA sequencing, Spared nerve injury

## Abstract

Monoamine-targeting antidepressants serve as frontline medications for chronic pain and associated comorbidities. While persistent anti-allodynic properties of antidepressants generally require weeks of treatment, several groups have demonstrated acute analgesic effects within hours of administration, suggesting a role in non-mesocorticolimbic pain processing regions such as the peripheral nervous system. To further explore this possibility, after four weeks of spared nerve injury or sham surgeries, we systemically administered desipramine or saline for an additional three weeks and performed whole transcriptome RNA sequencing on L3-6 dorsal root ganglia. Along with alterations in molecular pathways associated with neuronal activity, we observed a robust immunomodulatory transcriptional signature in the desipramine treated group. Cell subtype deconvolution predicted that these changes were associated with A- and C-fibers. Of note, differentially expressed genes from the dorsal root ganglia of DMI-treated, injured mice were largely unique compared to those from the nucleus accumbens of the same animals. These observations suggest that, under peripheral nerve injury conditions, desipramine induces specific gene expression changes across various regions of the nociceptive circuitry.

## Highlights

• Desipramine counteracts spared nerve injury-associated transcriptional signatures in the dorsal root ganglia.
• Desipramine induces an anti-inflammatory transcriptional signature in the dorsal root ganglia of nerve injured animals.
• Transcriptional effects of desipramine vary across pain processing regions after prolonged nerve injury.

## 1 Introduction

Chronic pain affects approximately 20% of adults in the United States (Yong et al., 2022) and is difficult to treat because of the diverse etiologies of the condition. One of the more successful treatment modalities for chronic pain has been the administration of antidepressants, including tricyclics such as desipramine (DMI). While the efficacy of antidepressants in treating chronic pain has largely been associated with the attenuation of maladaptations in higher pain processing circuits, such as the mesocorticolimbic system (Serafini et al., 2020), some recent studies have suggested additional therapeutic effects in peripheral nervous system regions such as the dorsal root ganglia (DRGs) (Kremer et al., 2016).

These system-wide effects may be largely due to the relative lack of target specificity that can result in various molecular cascades outside of monoamine regulation. For example, our group recently found that prolonged treatment with DMI one month after spared nerve injury (SNI) resulted in thousands of differentially expressed genes (DEGs) in the nucleus accumbens (NAc) (Serafini et al., 2023a). As such, we wanted to investigate the effects of prolonged desipramine treatment on transcriptional mechanisms within injured DRGs.

In this study, we performed RNA sequencing of DRGs from male mice treated with desipramine after prolonged peripheral nerve injury and demonstrated that DMI broadly counteracted SNI-induced gene expression signatures. To our surprise, pathways associated with DMI treatment were largely anti-inflammatory in nature and differed markedly from transcriptional signatures in the NAc of the same animals. These results suggest that, in addition to their known actions, tricyclic antidepressants also affect inflammatory pathways within the peripheral nervous system. It would be interesting to explore the efficacy of peripherally restricted analogs of existing antidepressants, which might reduce side effects such as vertigo and suicidality.

## 2 Materials and methods

### 2.1 Animals, Surgery, behavioral Assays, and drugs

3–4-month-old male C57BL6/J mice were used for this study in accordance with the Investigational Animal Use and Care Committee at the Icahn School of Medicine at Mount Sinai. Four animals were housed per cage with nesting materials at a controlled temperature of 70°F with 50% humidity, while on a 12-hour light/dark cycle.

Animals received either tibial spared nerve injury or sham surgery as previously described (Sakloth et al., 2020). Briefly, animals were placed under isoflurane inhaled anesthesia and the left hindleg was shaved and coated with 70% ethanol and betadine. The skin was incised and muscles were separated with forceps until the sciatic nerve was visible. The common peroneal and sural nerves were then ligated with a 6–0 silk suture and cut distally, after which an additional 1–2 mm segment of nerve was removed. Muscle was then closed with a 6–0 silk suture and skin was closed with a 4–0 nylon suture.

Four months after the injury surgery, animals received twice-daily intraperitoneal (i.p.) injections of either 15 mg/kg DMI in saline or saline alone for a period of three weeks. In order to behaviorally validate the lasting effects of desipramine treatment on injury-induced mechanical hypersensitivity, we used the von Frey assay > 16 hours after the previous treatment, in which the frequency of paw withdrawals was measured across filaments of various forces. Within each filament force, every mouse was tested once before re-testing a mouse for a total of five trials. The mechanical threshold was defined as the second force at which a filament elicited withdrawals in three of five applications. The experimenter was blinded during this assay. A repeated measures two-way ANOVA with Tukey’s post-hoc test on GraphPad Prism 10 was used to analyze von Frey data.

### 2.2 Tissue Preparation, RNA Sequencing, and bioinformatic analysis

L3-6 DRGs were harvested and flash frozen on dry ice. RNA was isolated using a TRIzol:Chloroform phase separation protocol as previously described (Avrampou et al., 2019). RNA libraries were generated using the Stranded mRNA Prep Kit (Illumina) and sequenced on the NovaSeq 6000 S1 Reagent Kit v1.5 (100 cycles) flow cell with 2x50bp paired-end reads.

FASTQ files were aligned to the mouse genome (mm10) using the NGS-Data-Charmer pipeline (https://github.com/shenlab-sinai/NGS-Data-Charmer) as previously described (Serafini et al., 2023a). DESeq2 (version 1.32.0) was implemented to identify any differential expression. Visualizations of the DESeq2 results (RRHO2 plots and volcano plots) were generated in R (version 4.1.1). Pathway analysis was performed using Ingenuity Pathway Analysis (IPA, Qiagen, Germany). Cutoffs of log2FC ≥ |0.32| and p-nominal < 0.05 were used for the identification of DEGs and downstream pathway/ontology analysis. Cell subtype deconvolution was performed using the CIBERSORTx tool as previously described (Newman et al., 2019, Pryce et al., 2023), with the signature matrix file constructed from the raw counts matrix of the single cell RNA sequencing dataset from a study by Renthal et al (Renthal et al., 2020). Multiple t-tests on GraphPad Prism 10 were used to analyze deconvolution results. Outliers from the deconvolution analysis were identified with the Grubb’s test (α = 0.05).

## 3 Results

In order to directly assess the effects of longitudinal antidepressant administration on DRGs that have been exposed to prolonged neuropathy, we administered desipramine (DMI) twice daily (15 mg/kg i.p.) for three weeks to male C57BL/6J mice that had received either SNI or sham surgery four weeks prior . The anti-allodynic efficacy of DMI by this timepoint was confirmed by von Frey in a recently published study by our group (n = 6 mice per condition; see Supplementary Materials) (Serafini et al., 2023a). L3 − 6 DRGs were harvested and processed for bulk RNA sequencing (n = 4 mice per condition).

**Fig. 1:**
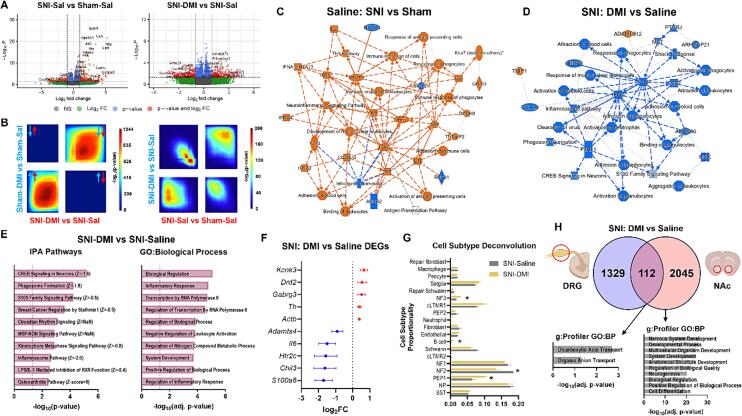
Transcriptional effects of DMI treatment on long-term nerve injury signatures. A) Volcano plots depicting DEGs (red) associated from the SNI-Saline vs Sham-Saline comparison (left) and the SNI-DMI vs SNI-Saline comparison (right). B) RRHO plots comparing SNI-DMI vs SNI-Saline and Sham-DMI vs Sham-Saline (left) and SNI-Sal vs Sham-Sal and SNI-DMI vs SNI-Saline (right). C & D) IPA summary graphs depicting major affected pathways and altered upstream regulator activity in the SNI-Saline vs Sham-Saline comparison (C) and in the SNI-DMI vs SNI-Saline comparison (D). E) Top IPA canonical pathways (left) and top GO:BP hits (right) in the SNI-DMI versus SNI-Saline comparison. F) Representative log2FC values for relevant DEGs from the SNI-DMI vs SNI-Saline comparison. G) Cell subtype deconvolution for the SNI-DMI vs SNI-Saline comparison (cLTMR1/2 = C-fiber low threshold mechanoreceptors, NF1/2/3 = A-fibers, NP = non-peptidergic nociceptors, PEP1/2 = peptidergic nociceptors, SST = pruriceptors; *p < 0.05 multiple t-tests). H) Comparison of DRG and NAc DEGs from the same animals in the SNI-DMI and SNI-Saline groups and accompanying g:Profiler GO:BP analysis. N = 3–4 animals per condition. Data expressed as mean ± SEM.

We identified 1,745 DEGs (572 p-adjusted < 0.1) in the SNI-Saline versus Sham-Saline comparison and 1,441 DEGs (106 p-adjusted < 0.1) in the SNI-DMI versus SNI-Saline comparison. Fig. 1**A** contains volcano plots depicting DEGs for the 1) SNI-Saline vs Sham-Saline and 2) SNI-DMI vs SNI-Saline groups. We next used rank-rank hypergeometric overlap (RRHO) to compare threshold-free gene expression changes between these groups. As seen in Fig. 1**B,** the Sham-DMI vs Sham-Saline and SNI-DMI versus SNI-Saline groups displayed a high degree of concordance, or a similar threshold-free regulation of genes. However, a discordant signature emerged when comparing the SNI-DMI versus SNI-Saline and SNI-Saline versus Sham-Saline groups **(**Fig. 1**B)**, suggesting a reversal of nerve injury signatures in the DRG after prolonged DMI treatment.

In order to investigate this further, we used Ingenuity Pathway Analysis (Qiagen) to characterize major molecular pathways affected by injury and DMI treatment. As seen in the IPA summary diagram in Fig. 1**C**, SNI alone induced a primarily pro-inflammatory transcriptional response, represented by a predicted activation of interleukins (IL1B, IL6, IL21, IL27) and interferons (IFNA1/2/4, IFNG, IFNL1) as upstream regulators, as well as activation of phagocytes, leukocytes, and antigen-presenting cells. IPA-generated upstream transcriptional regulators with predicted activity changes after SNI included STAT1 (activated, Z = 4.68, p = 7.14e-18), ZBTB10 (activated, Z = 4.997, p = 2.19e-16), JUN (activated, Z = 3.599, p = 1.79e-13), IRF2BP1 (inhibited, Z = -3.766, p = 3.6e-13), and CREB1 (activated, Z = 4.361, p = 4.8e-12), several of which have been implicated in neuropathic pain maintenance mechanisms in the DRGs (Barragán-Iglesias et al., 2020, Son et al., 2007, Yang et al., 2021).

Conversely, DMI treatment under SNI caused a predicted inhibition of IFNG and IL18 activity, while reducing neutrophil, granulocyte, leukocyte, and phagocyte activity **(**Fig. 1**D)**. Of note, these DMI-induced changes were also associated with a reduction in pain-associated pathways including CREB Signaling in Neurons, Phagosome Formation, and the Inflammasome Pathway (Fig. 1**E - left;** IPA Canonical Pathways). Gene ontology analysis confirmed the anti-inflammatory transcriptional effects of DMI treatment under SNI as seen by top GO: Biological Process pathways, such as Negative Regulation of Leukocyte Activation **(**Fig. 1**E - right).** IPA-generated transcriptional regulators with predicted activity changes in this condition included TCL1A (inhibited, Z = -2.236, p = 4.62e-6), ZFP36 (activated, Z = 2.985, p = 9.62e-6), IRF2BP2 (activated, Z = 3.199, p = 8.85e-5), BRD4 (inhibited, Z = -2.724, p = 1.66e-4), and CEBPE (inhibited, Z = -2.408, p = 3.25e-4).

However, DEGs from the SNI-DMI versus SNI-Saline comparison were not exclusively anti-inflammatory in nature. As seen in Fig. 1**F**, along with a reduction in pro-inflammatory genes (*Il6, Chil3, S100a8*), there were gene expression changes in serotonin (*Htr2c*), GABA (*Gabrg3*), and dopamine (*Drd2*) receptors, as well as changes in potassium regulation (*Kcnk3*), dopamine synthesis (*Th*), and cytoskeletal (*Actb*) and extracellular matrix (*Adamts4*) regulation. Surprisingly, cell subtype deconvolution analysis based on annotations from Renthal et al. (Renthal et al., 2020) suggested that DMI induced changes in A- and C-fiber-associated genes under SNI conditions, as opposed to injury-associated immune cell populations such as neutrophils and macrophages (Fig. 1**G**; multiple t-tests: PEP1 p = 0.0207, t-ratio = 3.117, df = 6; NF2 p = 0.0397, t-ratio = 2.763, df = 5; B cell p = 0.0335, t-ratio = 2.908, df = 5; NF3 p = 0.00137, t-ratio = 7.924, df = 4). Both SNI-DMI and SNI-Saline groups showed an increase in transcriptional contributions of macrophages relative to the Sham-Saline group (multiple t-tests: SNI-DMI vs Sham-Saline MeanDiff ± SEM = +0.0113 ± 0.00457, p = 0.049, t-ratio = 2.461, df = 6; SNI-Saline vs Sham Saline MeanDiff ± SEM = +0.0138 ± 0.00356, p = 0.0083, t-ratio = 3.867, df = 6).

We lastly wanted to determine whether any DMI-induced transcriptional effects were conserved across regions of the nociceptive circuitry by comparing DEGs between the DRG and NAc of the same animals. Interestingly, we identified only 112 genes that were differentially regulated in both pain-processing regions (NAc: 2,157 DEGs p-nominal < 0.05 & 298 p-adjusted < 0.1; Fig. 1**H**). These DEGs were largely unrelated, as the only significant ontologies associated with them were Dicarboxylic Acid Transport and Organic Anion Transport (g:Profiler GO:BP; Fig. 1**H**). Compared to inflammatory pathways observed with DRG DEGs from SNI-DMI animals, NAc DEGs were largely associated with neuronal development processes (g:Profiler GO:BP; Fig. 1**H**). Therefore, DMI appeared to have region-specific effects even under identical injury and treatment conditions.

## 4 Discussion

In this study, we identified a robust anti-inflammatory transcriptional signature in DRGs from nerve-injured mice after prolonged DMI treatment. This signature was largely different from that which was observed in the nucleus accumbens, suggesting region-specific mechanisms of action. While many groups have described the role of higher order pain regions in maintaining chronic pain states and comorbid affective abnormalities, the ability of antidepressants to attenuate peripheral inflammatory cascades might further explain the effectiveness of antidepressants in alleviating chronic pain. These findings might also further explain the acute anti-allodynic and anti-nociceptive properties of antidepressants that have been noted in several pre-clinical models.

Inflammation is a core mechanism underlying hypersensitivity of peripheral sensory neurons, as it is induced by various perturbations including nerve injury (Yu et al., 2020), viral infection (Serafini et al., 2023b), and chemical/diabetic (Hall et al., 2022, Zhang et al., 2016) neuropathies. Our findings were supported by Kremer et al., in which chronic duloxetine (a serotonin-specific reuptake inhibitor) administration alleviated chronic constriction injury-induced mechanical hypersensitivity and downregulated pro-inflammatory signatures, such as TNFα signaling (Kremer et al., 2018). When combined with our data, these findings suggest that structurally distinct monoamine-targeting antidepressants can attenuate injury immune responses across various neuropathy models. Furthermore, there is evidence that some, but not all, inflammation-associated effects of certain antidepressants are dependent on adrenergic signaling to the DRG (Kremer et al., 2018), and that β2-adrenoceptors (β2-ARs) are necessary for the anti-allodynic effects of DMI in neuropathy models (Yalcin et al., 2009). However, others have shown that inhibition of β2-AR activity can attenuate pain in various preclinical models (Shen et al., 2022). These findings also necessitate a consideration for the role of satellite glial cell (SGC) signaling in DMI therapeutic effects, as these cells express β2-ARs, demonstrate DMI-sensitive activity (Tang et al., 2010), and, upon activation, lead to neuronal hyperexcitability at least in part through pro-inflammatory cascades (Takeda et al., 2009). While our deconvolution suggested no changes in SGC-related DEGs, this could be due to the presence of various SGC subclasses that respond differently to injury states (Tonello et al., 2023). These considerations emphasize the importance of further work determining the effects of DMI on neuronal cells with and without the presence of non-neuronal cells.

The predicted alterations in upstream transcriptional regulator activity in DRGs from SNI-DMI animals added further color to the role of DMI in attenuating inflammatory-associated pathways from a neuronal perspective. For example, Hsieh et al. previously found that increased BRD4 expression in the DRG after complete Freund’s adjuvant was associated with activated Na_v_1.7 activity underlying hyperalgesia (Hsieh et al., 2017), and our analysis predicted an inhibition of BRD4 activity after DMI treatment. Furthermore, our deconvolution analysis predicted that the effects of DMI were associated with altered gene expression within neuronal subpopulations, suggesting a possible interruption of neuronal recruitment of circulating immune cells after nerve injury. While genes associated with broad classifications of infiltrating immune cells did not change between the SNI-DMI vs SNI-Saline groups, future work will delineate whether immune cell subtypes within these classifications are changing. For example, we observed a reduction in *Chil3* expression, an M2 macrophage marker which has been shown to decrease during recovery from nerve injury (Ydens et al., 2020). These findings emphasize the need to assess infiltrating immune cell signatures in the DRG after treatment with DMI over several timepoints and to implement analysis techniques that will provide insight on cell-type specific effects.

Of note, this study was limited by its exclusive use of male mice. In future studies, we will directly compare these signatures to a matched cohort of female mice. These findings will be important considering prior studies demonstrating sex specific changes in DRG injury pathways relevant to this study, such as TNFα signaling (Maguire et al., 2022) and TLR signaling (Chernov and Shubayev, 2021). Several of these sex differences observed in pre-clinical studies that are related to injury-induced inflammatory axes have been replicated in DRGs from neuropathic pain patients (Ray et al., 2023), further emphasizing the translational relevance of understanding the effects of antidepressants on peripheral nervous system injury responses.

## 5 Conclusions

DMI induced robust changes in gene expression in both the peripheral and central nervous system after prolonged administration. These changes appeared to be region-specific, and this may present an opportunity to more effectively treat complicated, chronic pain conditions that present with various maladaptations across the pain circuitry.

## Funding sources

This work was supported by NIH NINDS NS086444 and NS111351 to VZ.

## CRediT authorship contribution statement

**Randal A. Serafini:** Writing – original draft, Validation, Investigation, Formal analysis, Data curation, Conceptualization. **Aarthi Ramakrishnan:** Writing – review & editing, Methodology, Formal analysis, Data curation. **Li Shen:** Writing – review & editing, Supervision, Methodology, Data curation. **Venetia Zachariou:** Writing – review & editing, Supervision, Project administration, Funding acquisition, Conceptualization.

## Declaration of competing interest

The authors declare that they have no known competing financial interests or personal relationships that could have appeared to influence the work reported in this paper.

## Data Availability

RNA sequencing data is available on NCBI GEO under ID GSE261676.
