# Supplementary material for: Desipramine induces anti-inflammatory dorsal root ganglion transcriptional signatures in the murine spared nerve injury model
Source: Neurobiol Pain. 2024 Mar 20;15:100153. doi: 10.1016/j.ynpai.2024.100153 (PMC10973649; doi:10.1016/j.ynpai.2024.100153)
Supplement: Supplementary data 1 [file mmc1.docx]

**SUPPLEMENTARY DATA**

**
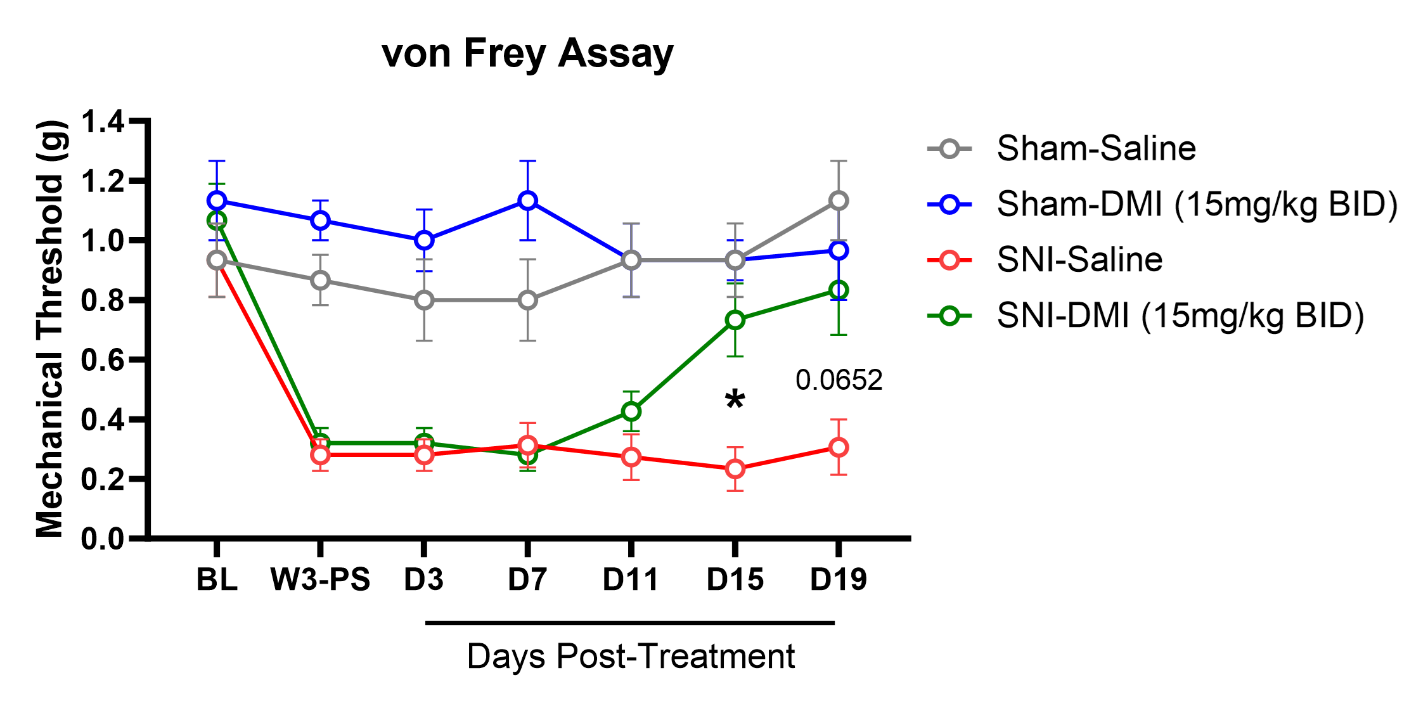
**

**Supplementary Figure 1. Desipramine alleviates mechanical hypersensitivity in male mice with prolonged peripheral nerve injury. A)** SNI-DMI mice showed significantly higher mechanical thresholds than SNI-Saline mice by 15 days after treatment initiation, which occurred on week four after surgery (n = 6 animals/group; repeated measures two-way ANOVA interaction factor F(18,120) = 2.756, p = 0.0005; Tukey’s m.c. SNI-Saline vs SNI-DMI D15 df = 8.159, q = 4.94, p = 0.0325; D19 df = 8.342, q = 4.227, p = 0.0652). Data are expressed as mean ± SEM. Abbreviations: W3-PS; Week 3 Post-Surgery; DMI = desipramine; BID = twice-daily.
